# Citrullinated glucose-regulated protein 78 is a candidate target for melanoma immunotherapy

**DOI:** 10.3389/fimmu.2022.1066185

**Published:** 2022-12-05

**Authors:** Victoria Anne Brentville, Peter Symonds, JiaXin Chua, Anne Skinner, Ian Daniels, Katherine Wendy Cook, Sasa Koncarevic, Roxana Martinez-Pinna, Sabaria Shah, Ruhul Hasan Choudhury, Poonam Vaghela, Daisy Weston, Abdullah Al-Omari, James Davis, Lindy G. Durrant

**Affiliations:** ^1^ Scancell Limited, Biodiscovery Institute, University of Nottingham, Nottingham, United Kingdom; ^2^ Proteome Sciences R & D GmbH & Co.KG, Frankfurt-am-Main, Germany; ^3^ Division of Cancer and Stem Cells, Biodiscovery Institute, University of Nottingham, University Park, Nottingham, United Kingdom

**Keywords:** citrullination, cancer, MHC-II, post-translation modifications, GRP78

## Abstract

**Introduction:**

Post translational modification of proteins plays a significant role in immune recognition. In particular the modification of arginine to citrulline which is mediated by PAD enzymes is increased during cellular stress (autophagy) which permits the presentation of modified epitopes upon MHC class II molecules for recognition by CD4 T cells. Citrullination also occurs in tumour cells as a result of continuous environmental stresses and increased autophagy. We have shown in animal models the efficient stimulation of citrullinated epitope specific CD4 T cells resulting in dramatic elimination/regression of tumours. The ER chaperone glucose-regulated protein 78 (GRP78) is known to also be required for stress-induced autophagy and is directly linked to autophagosome formation. GRP78 is known to be highly expressed by many tumour types. In this study we investigate the potential of targeting citrullinated GRP78 for cancer therapy.

**Methods:**

A citrullinated GRP78 specific antibody was used to assess citrullinated GRP78 expression in murine and human tumour cells by flow cytometry. Five peptides were selected and used to vaccinate HLA transgenic mice and immune responses were characterised by ex vivo cytokine ELISpot assay. T cell repertoire in humans was assessed through proliferation assays and cytokine ELISpot assay. Citrullinated peptide was identified in murine B16 melanoma by mass spectrometry and the peptide vaccine was assessed for tumour therapy in a mouse melanoma model.

**Results:**

We show the identification CD4 T cell responses to one citrullinated GRP78 epitope that are restricted through HLA DP*0401 and HLA-DR*0101 alleles. This peptide is detected by mass spectrometry in B16 melanoma grown in vivo and citrulline specific CD4 responses to two peptides spanning this epitope mediate efficient therapy of established B16 melanoma tumours in HHDII/DP4 (p<0.0001) transgenic mouse model. Finally, we demonstrate the existence of a repertoire of responses to the citrullinated GRP78 peptide in healthy individuals (p=0.0023) with 13/17 (76%) individuals showing a response to this peptide.

**Conclusion:**

We propose that citrullinated GRP78 is a candidate tumour antigen and vaccination against citrullinated GRP78 may provide a promising tumour therapy approach.

## Introduction

A major challenge for cancer immunotherapy is the stimulation of potent immune responses that can break tolerance and overcome the immune suppression of the tumour microenvironment. CD4 T cells are orchestrators of immune responses and have the capacity to influence the tumour environment through the release of pro-inflammatory cytokines such as IFNγ and TNFα as well as promotion of chemokine gradients that encourage infiltration of immune cells including antigen presenting cells (APCs), macrophages and CD8 T cells (1–4). In order for this to occur CD4 responses need to recognize antigen in the tumour environment. The tumour must therefore produce and present epitopes that have avoided thymic regulation and bind with higher affinity than other self-epitopes. This could be a neo-epitope which is formed by a mutation that occurs during oncogenesis (5, 6). However, this strategy is patient specific and is hampered by the heterogeneous nature and ongoing adaptation of tumours (7). An alternative way to overcome self-tolerance is to target post-translational modifications. The post-translational conversion of arginine residues to citrulline is mediated by peptidylarginine deiminase (PAD) enzymes (8, 9) which require micro molar levels of calcium to become activated (9). This can occur in double membrane vesicles such as the nucleus where citrullination alters gene regulation (10) or during autophagy when proteins are digested to provide additional energy in response to cellular stress (11). Citrullination alters protease cleavage resulting in different peptides being produced in stressed rather than healthy cells (12–14). If this process is also accompanied by inflammation and IFNγ secretion, then MHC class II (MHCII) molecules are upregulated on cells which can then present these stress related peptides (15). Subsequent recognition by cytotoxic CD4 T cells can result in the removal of these stressed or damaged cells. Cells undergo autophagy when experiencing stresses such as nutrient deprivation, oxygen deprivation, redox stress and DNA damage, to promote their survival. As such it is unsurprising that autophagy is upregulated in rapidly proliferating tumour cells (16). We have previously shown that vaccination with citrullinated epitopes from vimentin, a protein involved in epithelial to mesenchymal transition (EMT), enolase, a glycolytic enzyme, and a nuclear protein nucleophosmin generated cytotoxic CD4 T cells that rapidly eliminated tumours with no associated toxicity in mice (17–20). Citrullination has recently been proposed as a source of neoantigens in breast cancer with the detection of elevated anti-citrulline autoantibodies (21). During joint erosion associated with rheumatoid arthritis RA, apoptosis can lead to increased calcium levels, activation of PAD enzymes, citrullination and precipitation of proteins which are recognised and processed by B cells to produce an antibody response (reviewed in (22)). Subsequent presentation of epitopes to CD4 T cells results in affinity maturation and subclass switching of the antibody response. These antibodies can then form immune complexes within the joint exacerbating the disease and causing further inflammation. RA is associated with the shared epitope SE haplotypes and leading investigators to initially conclude that only these HLA molecules can present citrullinated epitopes (23). In contrast we and others have shown that alleles without the SE motif can present citrullinated epitopes (18, 24, 25) which is more in line with their role of recognising stressed cells in any individual. There is evidence that SE alleles may bind some citrullinated epitopes with higher affinity (26, 27) resulting in more aggressive activation of CD4 T cells which when accompanied by joint erosion, antibodies and inflammation can lead to RA.

The ER chaperone protein glucose-regulated protein 78 (GRP78) is a master regulator of ER stress and plays a vital role in protein assembly and targeting of misfolded proteins for degradation (28). ER stress triggered induction of GRP78 leads to enhanced survival of cancer cells and an association of GRP78 expression is linked to tumour progression (29–31). GRP78 has been shown to also be involved in calcium homeostasis and is required for stress induced autophagy (32). More recently citrullinated GRP78 has been defined as an autoantigen in Type 1 diabetes suggesting GRP78 is also a target for the PAD enzymes. Citrullinated GRP78 stimulates both antibody and T cell responses which have been detected in both rheumatoid arthritis and diabetes patients (33–35).

In this study we demonstrate in a murine model that citrullinated GRP78 is present in tumour samples and we identify a citrullinated GRP78 peptide which binds to and stimulates CD4 T cell responses restricted through multiple HLA alleles. Strong Th1 responses can be stimulated in HLA transgenic mice which lead to efficient tumour therapy of the aggressive B16 melanoma model. Furthermore, we show that the CD4 T cell repertoire to citrullinated GRP78 has not been deleted or tolerized and exists in healthy donors. We propose the targeting of citrullinated GRP78 as a new candidate for tumour therapy.

## Material and Methods

### Cell lines and culture

The murine melanoma B16F1, human MeWo melanoma, human SKOV3 and human MCF7 cell lines were obtained from the American Type Culture Collection (ATCC). SKOV3 and MCF7 cell lines were cultured in DMEM, high glucose or DMEM (Sigma) respectively, supplemented with 10% FCS. B16F1 and MeWo cell lines were cultured in RPMI medium 1640 (GIBCO/BRL) supplemented with 10% FCS, L-glutamine (2mM) and sodium bicarbonate buffered. B16F1 cell line was engineered with mouse MHC K/O and transfected with HHDII (HLA-A2) alone or in combination with either constitutive HLA-DP4 or IFNg inducible HLA-DP4 (iDP4) using plasmids as described previously (18). Cells were cultured in RPMI medium 1640 with L-glutamine (2 mmol/L), 10% FCS and appropriate antibiotics to maintain plasmids. Cell lines utilized were mycoplasma free, authenticated by suppliers (STR profiling), and used within ten passages. Cell lines utilized are certified mycoplasma free.

### Antibody generation and characterisation

Citrullinated GRP78 antibody was generated by immunization of Balb/c mice *via* intraperitoneal route with 50µg citrullinated GRP78 283-294 (BiPcit1) peptide linked to KLH (day1) and boosted with peptide linked to BSA (day 15) and Ova (day 29) all in combination with Titermax gold adjuvant (Sigma-Aldrich). Sera was screened for antibodies in ELISA at week 5-6 and positive mice were boosted at week 7 with peptide linked to KLH. A week after the boost splenocytes were fused with NS0 cells and hybridomas established by limiting dilution cloning. Antibody positivity and specificity was assessed by ELISA. ELISA for detection of peptide specific antibodies was performed by coating peptides onto high binding plates at 2.5µg/well in PBS overnight at 4°C. Plates were washed and blocked with 2% BSA/PBS followed by addition of purified antibody or hybridoma supernatant. Antibody binding was detected using anti-mouse HRP antibody and TMB core+ substrate (BioRad) with reaction stopped using 2M H_2_SO_4_ and absorbance read at 450nm.

### Staining of tumours

*In vitro* grown B16F1 and MeWo tumour lines untreated or treated for 5hrs at 37°C with 2µM thapsigargin (Sigma-Aldrich) or SKOV3 and MCF7 tumour lines were stained with citrullinated GRP78 antibody at 15µg/ml or isotype control followed by a FITC conjugated anti-mouse IgG secondary antibody (Sigma-Aldrich) in combination with a fixable live/dead stain (Thermofisher) and using intracellular fixation and permeabilization buffer set (Thermofisher). *In vivo* grown B16F1 tumours were mechanically disaggregated and stained as above with inclusion of surface stain for CD45 (anti-mouse CD45 efluor450, Thermofisher).

### Tumour lysate preparation and mass spectrometry analysis

For optimization of target peptide detection, a rat brain sample was lysed in 4M Gua*HCl 100 mM triethylammonium bicarbonate (TEAB) and protein concentration estimated using modified Bradford assay. Two aliquots, corresponding to ~40 mg protein equivalent, were spiked with 9 pmol and 900 pmol of the mixture of 20 peptides. Samples were labelled with TMTzero™ and subsequently processed through ultrafiltration cartridges (50 kDa cut-off, Amicon, Millipore). The flowthroughs were purified *via* solid-phase extraction on HLB-cartridges (Waters) and eluates split into and dried in a SpeedVac.

For tumour sample analysis, murine tumour lysates in 4M Gua*HCl 100 mM TEAB were assessed for protein concentration using modified Bradford assay. Four aliquots of the synthetic peptide mixture were reduced, alkylated and labelled with TMT™ reagents (TMT10-130N, TMT10-130C, TMT10-131, TMT11-131C) and mixed in a 1:4:6:10 ratio. 0.8 mg of murine protein from each of 5 tumour samples was TMT-labelled (TMT10-126, TMT10-127N, TMT10-127C, TMT10-128N, TMT10-128C) and mixed to form ~4 mg of murine protein mixture. The sample was processed through ultrafiltration cartridges (50 kDa cut-off, Amicon, Millipore) to enrich lower mass proteins and endogenous peptides in the flow-through. The sample was reduced, alkylated and spiked with 90 pmol of the synthetic peptide mixture (in a 1:4:6:10 ratio) before HLB purification. The resulting eluate was divided into two aliquots (10% unfractionated and 90% to bRP fractionation) and dried. The unfractionated sample was transferred to MS and bRP-fractionation was conducted using the Pierce High pH Reversed-Phase Peptide Fractionation Kit according to manufacturer`s instruction for TMT-labelled peptides to initially produce 8 fractions. Fractions 1 and 2 were combined as well as fractions 7 and 8 to finally produce six fractions.

Samples were analysed using an EASY-nLC™ 1000 system coupled to an Orbitrap Fusion™ Tribrid™ Mass Spectrometer (both Thermo Scientific). Re-suspended peptides were loaded onto a nanoViper C18 Acclaim PepMap 100 pre-column (Thermo Scientific) and resolved using an increasing gradient of ACN in 0.1% Formic acid through an 50 cm, 75 um ID analytical column with integrated emitter (Thermo Scientific) at a flow rate of 200 nL/min during the first 160 minutes and 300 nL/min over the last 20 minutes. LC gradient started with 5% up to 30% ACN or 10% up to 30% ACN over the first 160 minutes, and then 90% ACN over the last 20 minutes. Peptide spectra were acquired in data-dependent mode, with a 3-second duty cycle. Full spectra were acquired at 120K resolution, dependent MS scans at 30K resolution with a maximum fill time of 100 ms and an AGC target of 1e5. HCD fragmentation was performed at a normalized collision energy of 38. A second replicate was acquired using a neutral loss-triggered data-dependent acquisition. Full scan settings were as described above. CID fragmentation at a normalized collision energy of 30 was performed, followed by a low resolution scan in the ion trap analyzer. Detection of the neutral loss of isocyanic acid (diagnostic for presence of a citrullination triggered a dependent fragmentation scan in the Orbitrap after HCD fragmentation at a collision energy of 29. Simultaneously, an inclusion list of target peptide masses was used to trigger the fragmentation of prespecified target masses at an HCD of 30. All Orbitrap-based MS2 scans were acquired at 30K resolution, with a maximum injection time of 54ms, and an AGC target of 5e4.

Data files were searched against a murine non-redundant UniProtKB database (vs Oct 2020) supplemented with synthetic reference peptide sequences using Sequest in Proteome Discoverer 2.1. No cleavage specificity was enforced, precursor mass tolerance was set to 20 ppm, fragment mass tolerances were set to 0.6 or 0.02 Da for ion trap and Orbitrap scans, respectively. TMT modification of N-termini and lysines were, as well as carbamidomethylation of cysteines were set as static modifications, oxidation of methionine and citrullination of arginine were set as variable modifications. A 1% PSM-level false discovery rate was enforced *via* the Percolator algorithm (36). TMT reporter values were corrected for isotopic impurities and, subsequently, adjusted relative to the 131C (reference) channel and log_2_-transformed. For peptides with multiple (matched) spectra, peptide expression values were calculated as the median across all PSM values.

### Peptides and adjuvants

Peptides were synthesized at > 90% purity by Genscript (USA) and stored lyophilized at -80 °C. On the day of use they were reconstituted to the appropriate concentration in PBS. Adjuvants TLR9 agonist CpG ODN 1826 (*In vivo*gen) and TLR4 agonist MPLA (Sigma) were used at 5µg dose unless stated otherwise. TiterMax Gold was used at 100µl/dose.

### Immunisation protocol

Animal experiments were carried out with ethical approval under a Home Office approved project license. HLA-A2/DR1 (HHDII/DR1, Pasteur Institute), HLA-A2.1+/+ HLADP4+/+ hCD4+/+ (HHDII/DP4; EM:02221, European Mouse Mutant Archive) transgenic mice aged 8-12 weeks were used. For all studies the mice were randomized into different groups and not blinded to the investigators. Peptides were dissolved in PBS and then mixed with adjuvant and delivered at 10nmol dose unless stated otherwise. Peptides in adjuvant were injected subcutaneously at the base of the tail or intradermally unless stated otherwise. Mice were immunized on day 0, 7 and 14 and spleens were removed for analysis at day 20.

For tumour therapy experiments mice were challenged with 1x10^5^ B16 inducible HLA-DP4 cells or 5x10^5^ B16 cells expressing constitutive HLA-DP4 subcutaneously on the right flank 3 days prior to primary immunization and then were immunized as above. Tumour growth was monitored at 3-4 days intervals and mice were humanely euthanized once tumour reached 10-15 mm in diameter.

### *Ex vivo* ELISpot assay

Elispot assays were performed using murine IFNγ capture and detection reagents according to the manufacturer’s instructions (Mabtech). In brief, the IFNγ specific antibody were coated onto wells of 96-well Immobilin-P plate. Synthetic peptides (Genescript) at 10 µg/ml diluted in culture media (RPMI medium 1640 (GIBCO/BRL) supplemented with 10% FCS (Sigma), 2mM L-glutamine (Sigma) and sodium bicarbonate buffered with additional 20mM HEPES (Sigma) and 50µM 2-mercaptoethanol (Thermofisher)) and 5x10^5^ per well splenocytes were added to the wells of the plate in quadruplicate and plates incubated for 40 hrs at 37˚C. Cells with culture media only were added as negative control and 5µg/ml Lipopolysaccharide (LPS, Sigma) was used as a positive control. After incubation, captured IFNγ was detected by biotinylated specific IFNγ antibody and developed with a streptavidin alkaline phosphatase and chromogenic substrate. Spots were analyzed and counted using an automated plate reader (Cellular Technologies Ltd).

### Human samples

Experiments using human blood were carried out with ethical approval. Details of donors are given in **Supplementary Table 1**. Samples were obtained from healthy donors (ethics review: 161-1711) from University of Nottingham following University of Nottingham review and ethical approval. Informed consent was obtained from all subjects involved.

### Proliferation and restimulation assay on human PBMCs

Peripheral blood sample (approx. 50 mL) was drawn into lithium heparin tubes (Becton Dickinson). Samples were maintained at room temperature and processed immediately following venipuncture. PBMCs were isolated by density gradient centrifugation using Ficoll-Hypaque. Proliferation assay of PBMCs were performed immediately after PBMC isolation. The median number of PBMCs routinely derived from healthy donor samples was 1.29 × 10^6^ PBMC/mL whole blood (range: 0.6 × 10^6^ - 2.25 × 10^6/^mL). The median viability as assessed by trypan blue exclusion was 93% (range 90-95%). For CD25 depletion PBMCs were processed as above and then immediately depleted of CD25+ cells using anti-CD25 microbeads and MACS Cell Separation Columns (Miltenyi).

Freshly isolated CD25 depleted PBMCs were loaded with carboxyfluorescein succinimidyl ester (CFSE) (ThermoFisher). Briefly, a 50 µM stock solution in warm PBS was prepared from a master solution of 5mM in DMSO. CFSE was rapidly added to PBMCs (5 × 10^6^ cells/mL loading buffer (PBS with 5% v/v heat inactivated FCS)) to achieve a final concentration of 5µM. PBMCs were incubated at room temperature in the dark for 5 mins after which non-cellular incorporated CFSE was removed by washing twice with excess (× 10 v/v volumes) of loading buffer (300 g x 10 mins). Cells were made up in complete media to 1.5 × 10^6^/mL and plated and stimulated with media containing vehicle (negative control), PHA (positive control, final concentration 10 µg/mL) or peptides (10 µg/mL) as described above. On day 7-11, 500 µL of cells were removed from culture, washed in PBS and stained with 1:50 dilution of anti-CD4 (PE-Cy5 or efluor450, clone RPA-T4, ThermoFisher), anti-CD8 (efluor 450 or VioGreen, clone RPA-T8, ThermoFisher). Cells were washed and fixed and analysed on a MACSQuant 10 flow cytometer equipped with MACSQuant software version 2.8.168.16380 using stained vehicle stimulated controls to determine suitable gates.

For restimulation assays, PBMCs were cultured in media with IL-2, IL-7 and IL-15 cytokines (Peprotech) and 1 µg/ml of citrullinated GRP78 189-208 peptide. On day 10, cells were harvested. Human IFNγ ELISpot kits (Mabtech) were used with 1 x 10^5^ cells/well with 10 µg/ml peptide or PHA (10 µg/ml) positive control in quadruplicate wells. ELISpot plates were incubated at 37°C for 24 hours in an atmosphere of 5% CO2 and then developed following the manufacturer’s instructions. Spots were analyzed using an automated plate reader (Cellular Technologies Ltd).

### Statistical analysis

For calculation of linearity (mass spectrometry data), peptide logRatios were delogrithmised and the following model was fitted to evaluate the intercept and slope of the calibration curve:


Ratio (Theoretical) ~ Intercept + Slope * Ratio (Measured)

The coefficient of determination (R^2^) was used as a measure of the fit quality (linearity).

Comparative analysis of the proliferation assay results was performed by applying paired students t test with values of P calculated accordingly. Comparative analysis of the Elispot results were performed by applying ANOVA with values of P calculated accordingly. Comparison of tumour survival was assessed by Log Rank test using the Graphpad Prism software. p-values<0.05 were considered statistically significant

## Results

### Citrullinated GRP78 is a candidate target in tumours

**Figure 1:**
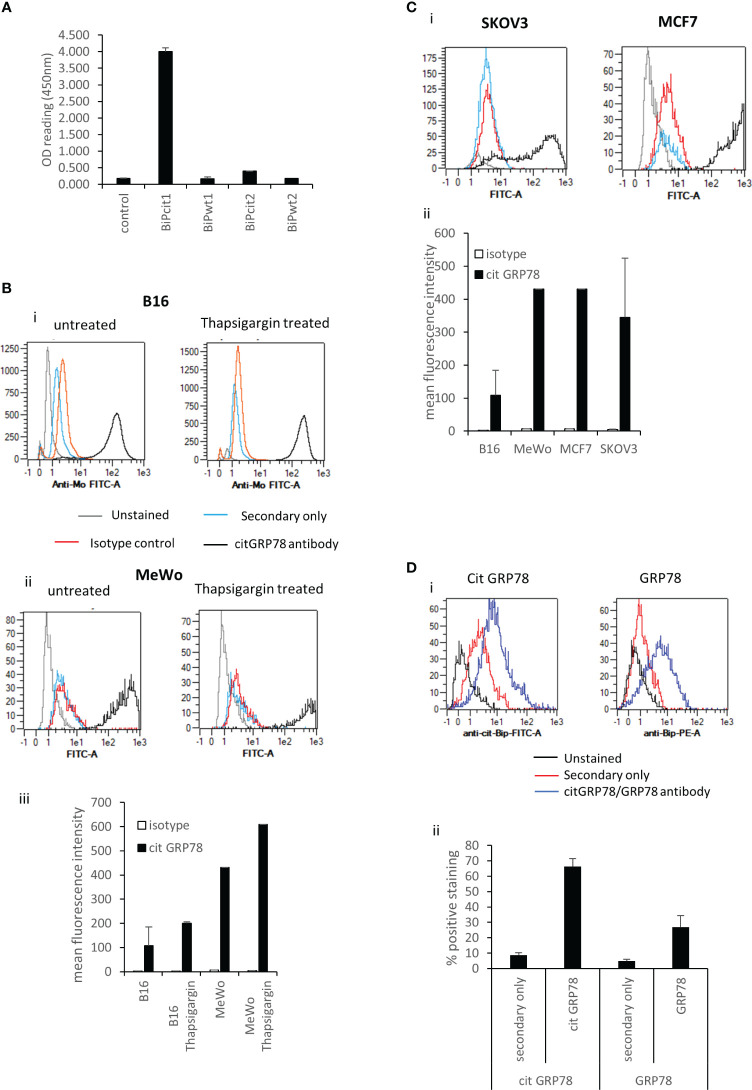
Citrullinated GRP78 is a candidate antigen in tumours. **(A)**, an antibody raised against citrullinated GRP78 peptide shows citrullinated GRP78 peptide specificity by ELISA. **(B)**, example staining of *in vitro* cultured B16 melanoma cells (i), MeWo melanoma cells (ii), untreated or treated with thapsigargin stained with citrullinated GRP78 antibody (black), secondary antibody only (blue), isotype control (red) or unstained (grey) and analysed by flow cytometry. (iii) collated data from at least two independent assays collated to show mean fluorescent intensity of citrullinated GRP78 antibody staining compared to isotype control. **(C)**, (i) example staining of *in vitro* cultured SKOV3 ovarian tumour cells or MCF7 breast tumour cells stained with citrullinated GRP78 antibody (black), secondary antibody only (blue), isotype control (red) or unstained (grey) and analysed by flow cytometry. (ii) collated data from at least two independent assays collated to show mean fluorescent intensity of citrullinated GRP78 antibody staining on B16, MeWo, MCF7 and SKOV3 cells compared to isotype control. **(D)**, Flow cytometry staining of B16 melanoma tumours ex vivo with citrullinated GRP78 or native GRP78 antibodies, (i) example staining plots, (ii) bar graph of percentage positive staining collated from at least four independent tumour samples.

To investigate if GRP78 is a target for citrullination in tumours we looked to identify citrullinated GRP78 in murine B16 tumours. This was firstly approached by using an antibody targeting a citrullinated GRP78 peptide. The antibody was generated against a citrullinated peptide from GRP78 (Bipcit1) that is homologous between human and mouse and was shown in ELISA to be specific for the citrullinated GRP78 and not the native sequence (BiPwt1) (**Figure 1A**). In addition, it did not react to an irrelevant citrullinated (Bipcit2) or corresponding native peptide (Bipwt2) from GRP78. The antibody was used to stain *in vitro* grown murine B16 melanoma cells and human melanoma line MeWo. Cells were stained with the citrullinated GRP78 antibody either untreated or treated for 5hrs with thapsigargin, an inducer of ER stress that is known to increase GRP78 expression. Untreated cells show staining with the citrullinated GRP78 antibody over that of isotype control (**Figure 1B**). Representative staining plots are shown in **Figures 1Bi, ii**. Collated data from replicate staining is shown in **Figure 1Biii**. The intensity of staining is increased in thapsigargin treated murine and human melanoma cells suggesting an increase in ER stress also leads to an increase in citrullinated GRP78 antibody staining although this does not reach significance (**Figure 1B**). To determine if staining with the citrullinated GRP78 antibody was specific to melanoma, the human SKOV3 ovarian and MCF7 breast cancer lines were also stained. These lines also demonstrate staining with the citrullinated GRP78 antibody *in vitro* (**Figure 1C**). Murine B16 melanoma tumours were subsequently grown in mice and, to confirm if the citrullinated GRP78 antibody could detect citrullinated protein *in vivo*, tumours were stained with the citrullinated GRP78 or a native GRP78 antibody directly *ex vivo*. **Figure 1Di** shows an example staining of *in vivo* grown B16 tumour. Staining was performed in the presence of Fc blocking reagent and gating was set to exclude dead cells and analyse only non-lymphoid CD45 negative cells. Gating strategy is shown in **Supplementary Figure 1**. **Figure 1Dii** shows collated data from four tumour samples stained with secondary only, citrullinated GRP78 or GRP78 antibodies. The graph shows the percentage positive cell staining gated on live CD45 negative cells where the positive gate is set on the unstained control. *Ex vivo* stained tumours show evidence of positive staining with both native GRP78 and citrullinated GRP78 antibodies suggesting that citrullinated GRP78 is expressed in tumours *in vivo* and could be a target for immunotherapy.

### Citrullinated GRP78 specific CD4 Th1 responses restricted through multiple HLA alleles can be stimulated in HLA transgenic mice

To confirm if citrullinated GRP78 is expressed in tumours and can be a target for immunotherapy, we sought to generate T cell responses specific for citrullinated GRP78. Citrullinated GRP78 is an autoantigen in rheumatoid arthritis (RA) which is known to be associated with the HLA SE alleles as well as in type 1 diabetes and CD4 T cells reactive to citrullinated GRP78 have been identified. CD4 T cell repertoires in HLA-DR*0401 positive autoimmune patients have been identified to both citrullinated and native GRP78 peptides (35). To determine if citrullinated GRP78 could be a target for CD4 T cells in tumour therapy we sought to identify citrulline specific responses restricted through two other HLA alleles. The GRP78 protein sequence was subject to analysis *via* the IEDB prediction software to identify potential peptides with high binding affinity to the HLA-DR*0101, an HLA-SE allele and HLA-DP*0401. Five peptides were chosen that were 1) not specifically described to be preferential CD4 T cell targets in autoimmune disease, 2) contained an arginine within the predicted binding core region and 3) showed a range of predicted binding affinities (**Table 1**). Only one peptide (aa189-208) was predicted to bind to HLA-DR*0101 with moderate affinity (**Table 1**). One of the five selected peptides, aa315-334, showed high predicted binding affinity to DP*0401.

**Table 1:** Peptide sequences and IEDB predicted binding scores.

Coordinates	Sequence	DR*0101 binding score	DR*0101 predicted cores	DP*0401 binding score	DP*0401 predicted cores
189-208	TIAGLNVM**R**IINEPTAAAIA	7.28	VM**R**IINEPT IAGLNVM**R**I	59.16	AGLNVM**R**II IAGLNVM**R**I LNVM**R**IINE
315-334	GEDFSETLT**R**AKFEELNMDL	54.2	SETLT**R**AKF FSETLT**R**AK LT**R**AKFEEL	0.13	LT**R**AKFEEL
327-346	FEELNMDLF**R**STMKPVQKVL	28.99	F**R**STMKPVQ LF**R**STMKPV MDLF**R**STMK	19.99	LF**R**STMKPV LNMDLF**R**ST
460-478	TVTIKVYEGE**R**PL*T*KDNHL	44.67	KVYEGE**R**PL YEGE**R**PL*T*K	17.85	KVYEGE**R**PL YEGE**R**PL*T*K
501-520	FEIDVNGIL**R**VTAEDKGTGN	35.86	IDVNGIL**R**V IL**R**VTAEDK	59.87	DVNGIL**R**VT IDVNGIL**R**V

Bold, arginine.

Italics, change between human and murine sequence.

**Figure 2:**
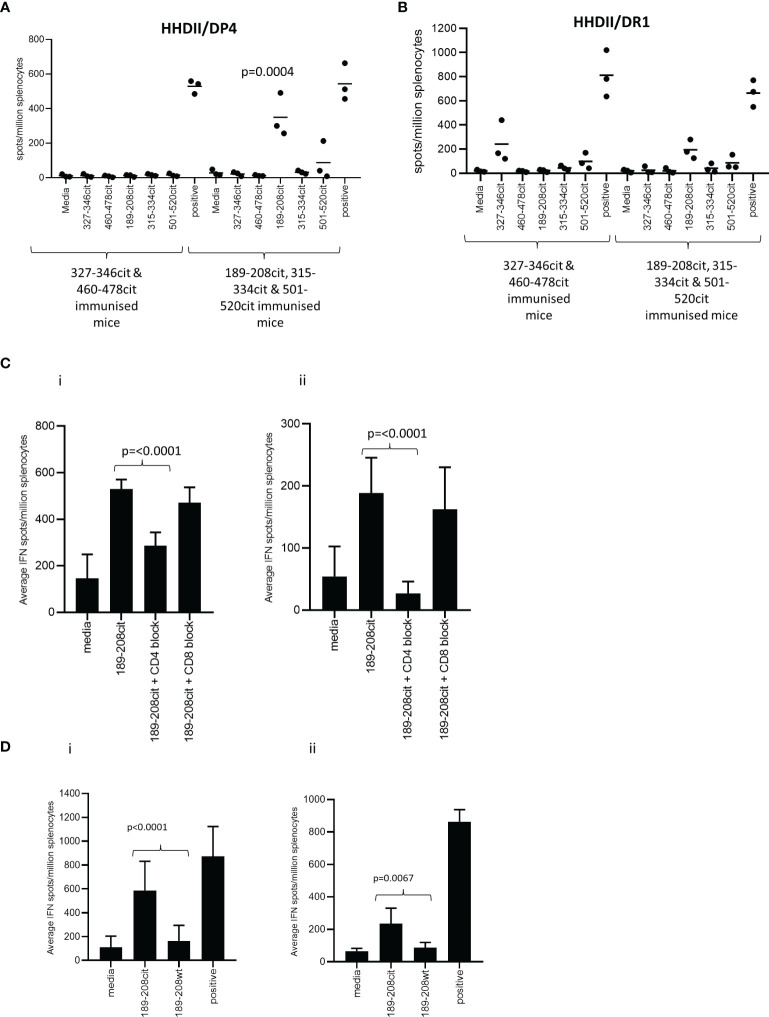
CD4 Th1 responses to citrullinated GRP78 can be detected in HLA transgenic mice. HHDII/DP4 **(A)** or HHDII/DR1 **(B)** transgenic mice were immunised with pools of citrullinated GRP78 peptides and responses measured to specific peptides by IFNg ELISpot assay. Responses in HHDII/DP4 (i) or HHDII/DR1 (ii) transgenic mice to citrullinated 189-208 peptide were assessed in the presence of CD4 or CD8 blocking antibodies **(C)** or for cross reactivity to the native 189-208 sequence **(D)** by IFNg ELISpot assay. Results are representative of at least two independent experiments in which n=3.

Predicted binding is based upon the native sequence and does not take account of the citrulline modification. Modification of a positive charged arginine to a neutral citrulline can alter structure and protein cleavage and subsequently affect peptide presentation by HLA molecules. Therefore, the five peptides were synthesised with citrulline in replacement for arginine and the citrulline containing peptides tested for immunogenicity in HLA-DR*0101 (HHDII/DR1) and HLA-DP*0401 (HHDII/DP4) transgenic mice. Peptide sequences are homologous between the human and mouse protein sequences with the exception of 460-478 which has a T to R change from human to mouse at amino acid 472. The five peptides were divided into two pools of non-overlapping sequences and used to immunise the mice. Responses were screened in IFNγ Elispot assay for reactivity to all five citrullinated peptides. Screening of responses in the HLA-DP*0401 transgenic mice showed strong responses to the citrullinated 189-208 peptide (p=0.0004) despite the native sequence having low predicted binding to the HLA-DP*0401 allele (**Figure 2A**). Low responses were also seen to the citrullinated 501-520 peptide in HHDII/DP4 transgenic mice although this did not reach significance. In addition to showing responses restricted through DP*0401 we also examined responses in the HHDII/DR1 transgenic mice. Out of the five peptides only aa189-208 was predicted to bind with moderate affinity to HLA-DR*0101. HHDII/DR1 transgenic mice were immunised with the citrullinated peptide pools and responses screened by IFNγ Elispot. HHDII/DR1 mice show low frequency responses to this peptide and the 327-346 peptide but these do not quite reach significance (**Figure 2B**). This suggests that the 189-208 peptide contains epitopes that may be restricted through a number of HLA alleles. This peptide was chosen for further studies.

The HHDII/DP4 and HHDII/DR1 transgenic mice in this study both also express the HHDII allele (human HLA-A2) therefore in order to confirm the MHC class II restriction, responses were assessed in the presence of CD4 and CD8 blocking antibodies. In the HHDII/DP4 transgenic strain responses were significantly reduced (p=0.0012) but not eliminated in the presence of CD4 blocking antibody suggesting a CD4 mediated (DP*0401 restricted) response (**Figure 2Ci**). The CD8 blocking antibody had no effect indicating no role for CD8 cells in this peptide specific response. In the HHDII/DR1 transgenic strain a similar observation is made for responses to the citrullinated 189-208 peptide with responses lost in the presence of CD4 blocking antibody but not in the presence of the CD8 blocking antibody (**Figure 2Cii**). This data suggests that responses are CD4 mediated in both these mouse strains.

In the HHDII/DP4 mice the response stimulated by the citrullinated 189-208 peptide was also shown to cross react with the 187-206 sequence (**Supplementary Figure 2A**). Immunisation with the citrullinated 187-206 sequence stimulated similar responses to those induced by the citrullinated 189-208 sequence (**Supplementary Figure 2B**) suggesting the core epitope for this HLA allele lies between aa 189-206.

To determine if the responses generated in the transgenic mice were specific to the citrulline modification, the responses stimulated by the citrullinated 189-208 peptide were tested for cross reactivity to the unmodified (wt) sequence. **Figure 2Di** shows the responses stimulated by citrullinated 189-208 peptide in the HHDII/DP4 mice show minimal cross reactivity to the wt peptide sequence. The response to the citrullinated peptide is significantly higher than that to the wt peptide (p<0.0001). A similar finding is seen in the HHDII/DR1 mouse model with the responses showing minimal cross reactivity to the wt peptide (p=0.0067) (**Figure 2Dii**).

### Citrullinated GRP78 189-208 can be detected in tumours

Since the citrullinated GRP78 189-208 peptide is capable of stimulating citrulline specific T cell responses through multiple HLA alleles we sought to confirm the presence of citrullinated GRP78 189-208 peptide in tumours before examining if it could be a target for tumour therapy. Lysates of B16 melanomas grown *in vivo* were analysed by targeted mass spectrometry for the presence of the citrullinated GRP78 189-208 peptide. Targeted detection of specific citrullinated peptide was optimised *via* spiking irrelevant brain protein preps with synthetic citrullinated peptide sequences. Following this 5 independent B16 tumour samples were analysed for presence of the citrullinated peptide with the use of synthetic peptide as a trigger for the determination of peptide abundance. 10,539 peptide sequences were detected within 2,151 protein groups. Citrullinated GRP78 189-208 peptide was detected with linearity value (R^2^) of greater than 0.90 and compared to detection of two vimentin and one alpha-enolase peptides known to be CD4 T cell targets in the same tumour model (17, 19, 37). The citrullinated GRP78 189-208 peptide showed the highest abundance value (expressed in pmol/mg total protein) (**Table 2**). Fragmentation MS spectra identifying the citrullinated GRP78 189-208 peptide are shown in **Supplementary Figure 3**.

**Table 2:** Targeted detection of citrullinated peptides by mass spectrometry in B16 tumour lysates.

Antigen	Peptide coordinates	Sequence	Linearity (R^2^)	Average pmol/mg
GRP78	189-208	TIAGLNVM-cit-IINEPTAAAIA	0.93	3.086
Vimentin	415-433	LPTFSSLNL-cit-ETNLESLPL	0.90	1.704
Vimentin	28-49	cit-SYVTTST-cit-TYSLGSAL-cit-PSTS	1.00	0.856
Alpha-enolase	241-260	vigmdvaasefY-cit-sgkydld	0.97	0.704

cit, citrulline.

### Immunisation with citrullinated GRP78 189-208 peptide elicits tumour therapy

**Figure 3:**
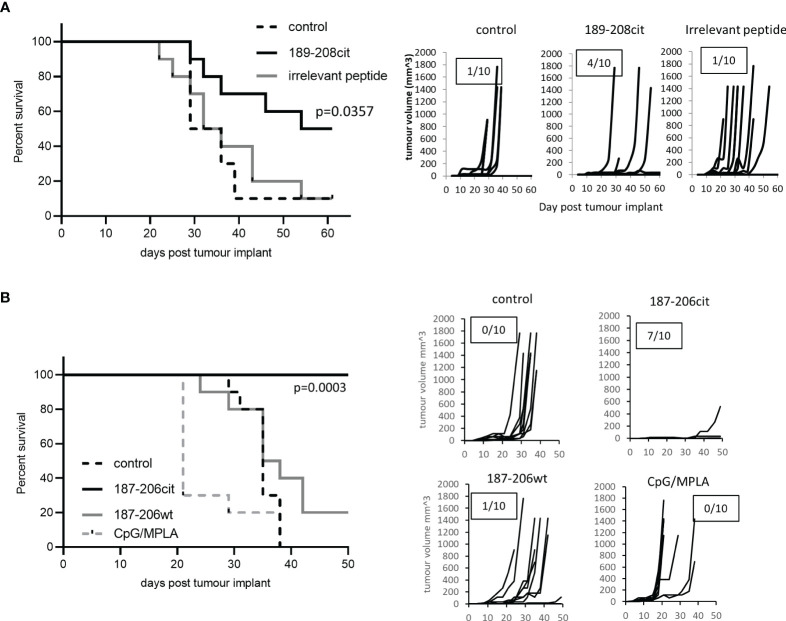
Citrullinated GRP78 peptide vaccination mediates efficient tumour therapy. **(A)** HLA-DP4 mice were challenged with B16 cells constitutively expressing HLA-DP*0401 and four days later immunised with citrullinated GRP78 189-208 peptide or an irrelevant peptide. **(B)**, HHDII/DP4 transgenic mice were challenged with B16 cells expressing HLA-DP*0401 under an IFNγ inducible promoter. Four days later mice were immunised citrullinated (cit) or native (wt) GRP78 peptide or adjuvant only control and tumour growth and survival monitored. N=10/group. Survival curves and tumour growth curves are shown. Numbers in boxes on the tumour growth curve graphs represent the number of tumour free animals out of the total animals in each group.

We have shown that the citrullinated 189-208 peptide can be presented on MHC class II for recognition by T cells and is present in lysate from B16 tumours. To assess if the T cell responses were capable of responding to the peptide presented in the tumour environment *in vivo* and provide tumour therapy, we assessed them for therapeutic anti-tumour effect. HHDII/DP4 mice were implanted with B16 melanoma constitutively expressing DP*0401 and HHDII but lacking any murine MHC class I or II. Mice were immunised with the citrullinated 189-208 peptide and tumour growth and survival monitored. **Figure 3A** shows a significant enhancement of survival and delayed tumour growth with 50% survival at day 60 compared to irrelevant peptide immunised mice (p=0.0357). Numbers shown on the tumour growth curves represent the number of tumour free mice with 4 of 10 mice in the citrullinated 189-208 peptide group remaining tumour free. The citrullinated GRP78 189-208 peptide has been shown to stimulate responses that cross react with the 187-206 sequence therefore this sequence was also assessed for a therapeutic anti-tumour effect. Tumours *in vivo* do not often constitutively express MHC class II therefore the GRP78 187-206 citrullinated peptide was assessed for tumour therapy in a tumour model where HLA-DP*0401 is expressed under an IFNγ inducible promoter. Despite this model being marginally less immunogenic than the model expressing constitutive HLA-DP*0401, **Figure 3B** shows significantly enhanced survival with 100% of mice treated with the citrullinated peptide surviving to day 50 and 7 of 10 being tumour free compared to unimmunised or adjuvant only controls (p<0.0001) and those receiving the wild type peptide (p=0.0003). This data suggests that the citrullinated epitope within GRP78 189-208 and 187-206 sequences is a target for specific CD4 T cells in tumours and can be efficiently targeted for tumour therapy.

### Healthy humans show a repertoire of CD4 T cells able to respond to citrullinated GRP78 189-208 peptide

**Figure 4:**
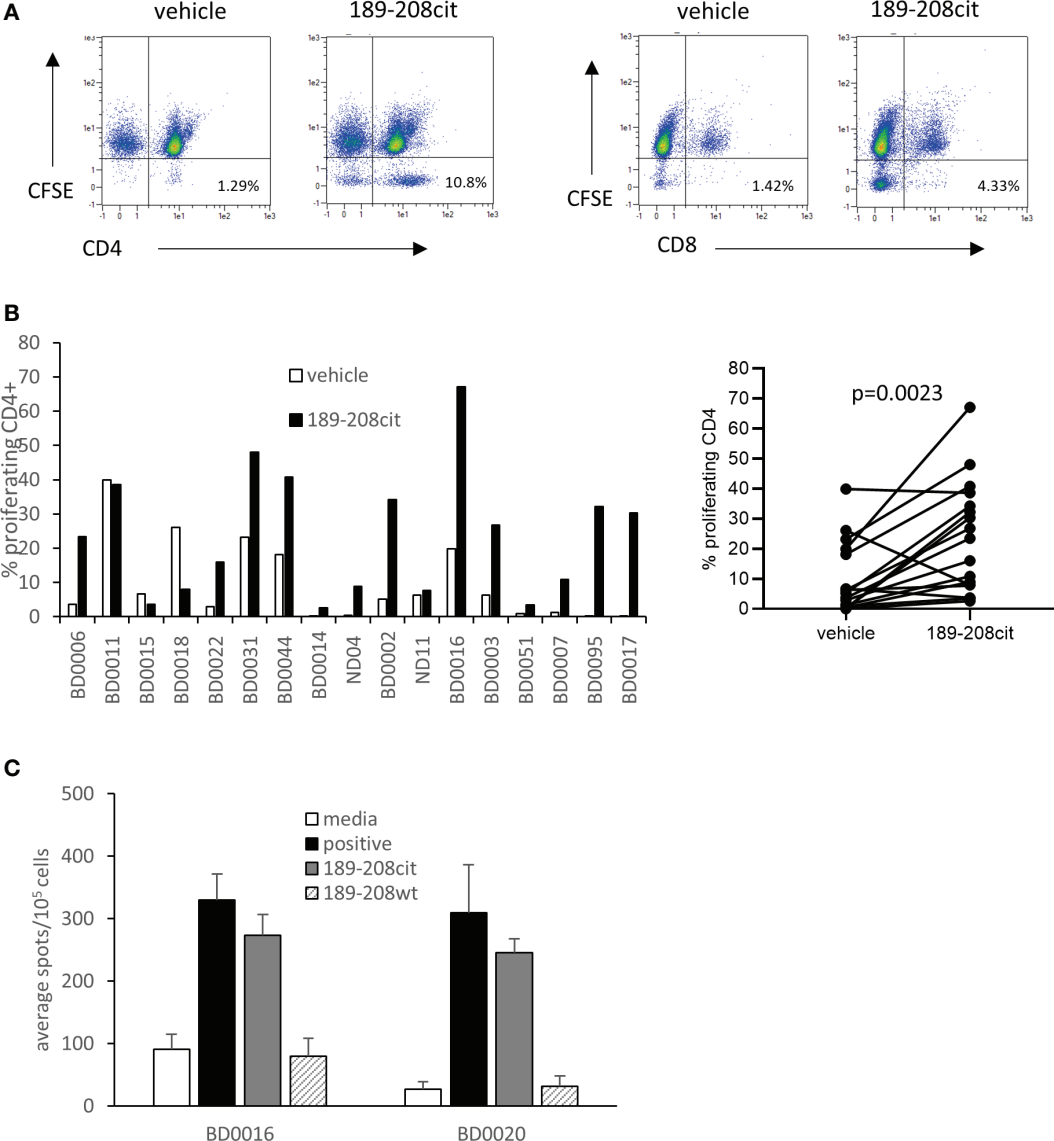
A CD4 T cell repertoire to citrullinated GRP78 peptide is detected in healthy donors. **(A)**, an example staining profile of healthy donor PBMCs responding to citrullinated GRP78 189-208 peptide. Numbers represent percentage of CFSE low CD4+ or CD8+ cells. **(B)**, A panel of healthy donor PBMCs were analysed for proliferation in response to citrullinated GRP78 189-208 peptide. Results shown as percentage proliferating CD4+ cells shown for each donor (i) and combined (ii). **(C)**, IFNγ ELISpot on PBMC cultures from two donors for restimulation responses to citrullinated or native (wt) GRP78 189-208 peptide.

We have previously shown that CD4 responses to citrullinated peptides can be detected in healthy donors and cancer patients. Here we examine the responses in seventeen healthy donors to citrullinated GRP78 189-208 peptide. Responses were measured by proliferation using CFSE dilution assay at day 10 and deemed positive if above twice background and greater than 1%. Example responses at day 7 and day 10 post stimulation compared to a known peptide specific CD4 response and gating strategy is shown in **Supplementary Figure 4**. Responses were shown to be CD4 mediated as shown in an example from donor BD007 (**Figure 4A**). In total thirteen of seventeen donors showed a CD4 proliferative response to the citrullinated GRP78 189-208 peptide and overall, a significant response (p=0.0023) to the citrullinated 189-208 peptide was observed (**Figure 4B**). To determine if responses were modification specific restimulation assays were performed on two donors where PBMCs had been cultured with citrullinated GRP78 189-208 peptide for 10 days. IFNƴ ELISpot assays demonstrated restimulation responses to the citrullinated peptide that did not cross react with the native (wt) sequence (**Figure 4C**) suggesting that the T cells stimulated were specific to the modification. Where HLA typing was available responding donors appeared to share the HLA-DP*04 allele in common (**Supplementary Table 1**) confirming this to perhaps be a restricting allele in the healthy human population although further characterisation would be required to confirm this. However, this data confirms that humans have a modification specific repertoire that can respond to citrullinated GRP78 189-208 epitope.

This study provides evidence for the expression of citrullinated GRP78 in tumours and that T cell responses to citrullinated GRP78 have not been subject to thymic tolerance in mice models and in humans. This data demonstrates the potential of citrullinated GRP78 as a new target antigen for tumour therapy.

## Discussion

The ER chaperone GRP78 is known to be overexpressed in many cancer types and has been associated with poor prognosis and higher pathological grade (29, 38, 39). GRP78 is a known target of citrullination (32) and given the association of GRP78 with ER stress it is possible citrullination of GRP78 occurs during such times of cellular stress when intracellular Ca^2+^ levels are elevated. Citrullinated proteins have been detected in tumour cells as well as in autoimmune disorders and can be targeted for tumour immunotherapy (17, 19, 20, 37, 40). In murine models of ovarian cancer, tumour ascites express GRP78 on the cell surface and over expression of GRP78 in ovarian tumour cells as well as other tumour types correlates with malignant transformation and cancer progression (29, 41–43). In this report we generated an antibody recognising citrullinated GRP78 but not the native protein to help establish the expression of citrullinated GRP78 in tumours. Staining of the murine B16 melanoma cell line and three human tumour cells lines with the citrullinated GRP78 antibody was seen under normal *in vitro* growth conditions. Since GRP78 is an ER resident protein and the ER is known to have higher calcium concentrations than the cytosol (44), it is perhaps not surprising that some citrullinated GRP78 may be present under normal conditions. However, in the absence of inflammation and MHCII expression it is unlikely to become a target for T cells. Treatment with thapsigargin, an ER stress inducer known to increase GRP78 expression levels (45), led to higher staining levels *in vitro* suggesting that ER stress may increase citrullinated GRP78 levels. *In vivo*, the combination of ER stress and other cellular stresses have the potential to both increase GRP78 expression and enhance autophagy to increase citrullination but in the absence of inflammation, MHCII peptide presentation would be limited. Expression of citrullinated GRP78 *in vivo* was confirmed through the detection of the citrullinated GRP78 189-208 peptide by mass spectrometry in tumour lysates and this was demonstrated at a level greater than other citrullinated epitopes already shown to be targets in B16 melanoma (17–19). This provides a rationale for citrullinated GRP78 as a candidate target for tumour therapy.

In this study we selected and screened a number of citrullinated GRP78 peptides for binding to two HLA alleles, HLA-DR*0101 and HLA-DP*0401, other than HLA-DR*0401. Our previous work and others have also shown that citrullinated peptides have affinity for other non-SE alleles and we again confirm this showing CD4 T cell responses to citrullinated GRP78 peptides in HHDII/DP4 transgenic mice (18, 24, 25). Immune response analysis in HLA transgenic mice identified one peptide (aa189-208) that stimulated IFNγ responses across two HLA alleles, HLA-DP*0401 and HLA-DR*0101 and this was shown to cross react with a peptide spanning aa187-206. Characterisation of these responses revealed that they were CD4 mediated and citrulline specific suggesting a distinct T cell repertoire. The citrullinated GRP78 189-208 and 187-206 specific CD4 responses in the mouse models were used to confirm the presence of citrullinated GRP78 in tumours and its relevance as a target for tumour therapy. CD4 responses have been shown to have an increasingly important role in anti-cancer immunity (46, 47) and our work and that of others has shown that CD4 responses can directly mediate tumour therapy in mouse models (17, 19, 20, 37, 48). The strong Th1 responses to citrullinated GRP78 peptide provided efficient therapeutic responses compared to the native peptide in the HHDII/DP4 tumour model. Anti-tumour immunity was observed against B16 tumours expressing either constitutive or IFNγ inducible HLA-DP*0401 suggesting that the CD4 T cell response may exert both direct and indirect effects upon the tumour to promote inflammation and MHCII upregulation. In addition to direct recognition of tumours, CD4 T cells can also be involved in the activation of M1 macrophages and provision of help for CD8 T cells (49, 50). We demonstrate the efficacy of a vaccine mediated approach to cancer therapy targeting the citrullinated GRP78 peptide. However, other approaches such as the generation of bispecific antibodies simultaneously targeting CD3 and a peptide/MHC complex or combining CD3 and a specific TCR like binding domain recognising peptide/MHC complex have shown efficacy in preclinical studies and could also be considered (51–53).

The stimulation of citrulline specific T cell responses has the potential for on target/off tumour effects specifically on inflamed tissues and may exacerbate autoimmunity. In spite of this possibility no toxicity was observed in our murine studies up to 50 days post immunization. Although T cells can respond to citrullinated peptides, the same peptides are unlikely to stimulate antibody responses, and both are needed to cause autoimmune diseases such as RA. This is exemplified in this study and others in mouse models where no autoimmune effects are observed with T cells alone, even in models susceptible to RA (17, 19, 54). However, the possibility of exacerbating autoimmune disease through stimulating citrulline specific T cell responses should not be underestimated, and signs of autoimmunity should be monitored especially in patients with underlying autoimmunity and these individuals should be excluded from any future clinical trial.

Analysis of responses to citrullinated GRP78 189-208 in humans revealed the detection of CD4 responses in 76% healthy donors indicating a T cell repertoire exists to this epitope that has not been subject to central tolerance or deletion. This repertoire was demonstrated to be specific for the citrulline modification. Responding donors expressed a variety of HLA class II types with HLA-DP*0401, an allele expressed by 70% of the Caucasian population, common among all responding donors where HLA typing was available. Cancer can influence the T cell response repertoire therefore it would be interesting in future studies to compare similar responses in cancer patients to examine if the cancer has any impact on the responses that can be stimulated. We propose that citrullinated GRP78 is a candidate tumour antigen and vaccination against citrullinated GRP78 may provide a promising tumour therapy approach.

## Data availability statement

The datasets presented in this study can be found in online repositories. The names of the repository/repositories and accession number(s) can be found below: PRIDE repository *via* accession ID: PXD037918.

## Ethics statement

The studies involving human participants were reviewed and approved by University of Nottingham review and ethical approval (ethics review: 161-1711). The patients/participants provided their written informed consent to participate in this study. The animal study was reviewed and approved by Nottingham Trent University Animal Welfare and Ethical Review Board and Nottingham University Animal Welfare and Ethical Review Board.

## Author contributions

LGD and VAB directed the study. PS, JC, AS, ID, SS, KWC, SK, RM-P, RHC, PV, DW, AA and JD performed experiments and analyzed data. VAB designed experiments, analyzed the data and wrote the paper. All authors contributed to the article and approved the submitted version.

## Funding

The authors declare that this study received funding from Scancell Ltd. The funder had the following involvement with the study: study design, collection, analysis, interpretation of data, the writing of this article.

## Acknowledgments

The authors would also like to thank Dr Samantha Paston and Dr Mireille Vankemmelbeke for help in proofreading the manuscript.

## Conflict of interest

Authors SK, RMP were employed by Proteome Sciences R & D GmbH & Co.KG. LD is a director and shareholder in Scancell Ltd.

The remaining authors are employees of Scancell Ltd.

## Publisher’s note

All claims expressed in this article are solely those of the authors and do not necessarily represent those of their affiliated organizations, or those of the publisher, the editors and the reviewers. Any product that may be evaluated in this article, or claim that may be made by its manufacturer, is not guaranteed or endorsed by the publisher.
