# Supplementary material for: Citrullinated glucose-regulated protein 78 is a candidate target for melanoma immunotherapy
Source: Front Immunol. 2022 Dec 5;13:1066185. doi: 10.3389/fimmu.2022.1066185 (PMC9760948; doi:10.3389/fimmu.2022.1066185)
Supplement: Supplementary file 1 [file DataSheet_1.docx]

Supplementary Material

# Supplementary Tables

##
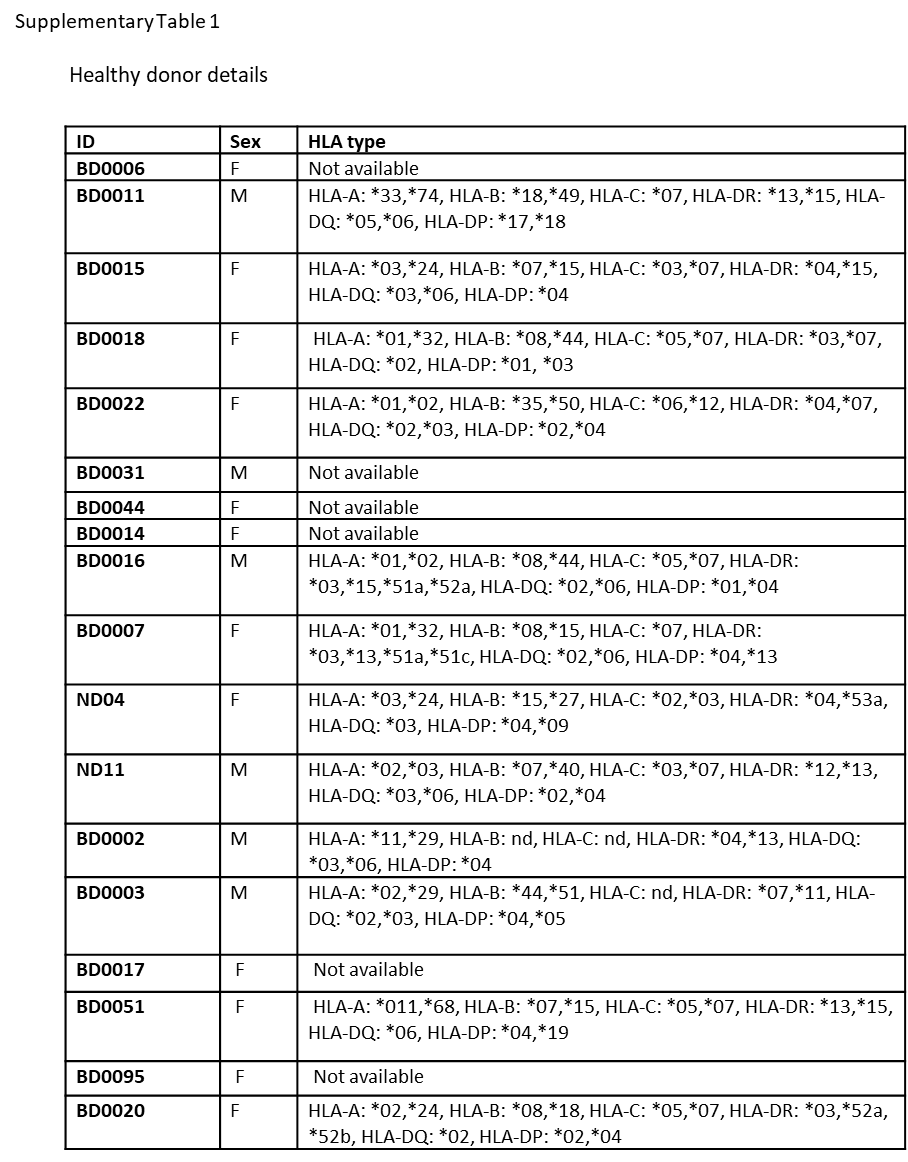


# Supplementary Figures

Supplementary Figure 1.


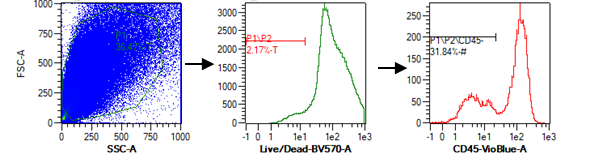


Gating strategy for staining of ex vivo tumors with citrullinated GRP78 antibody

Supplementary Figure 2.


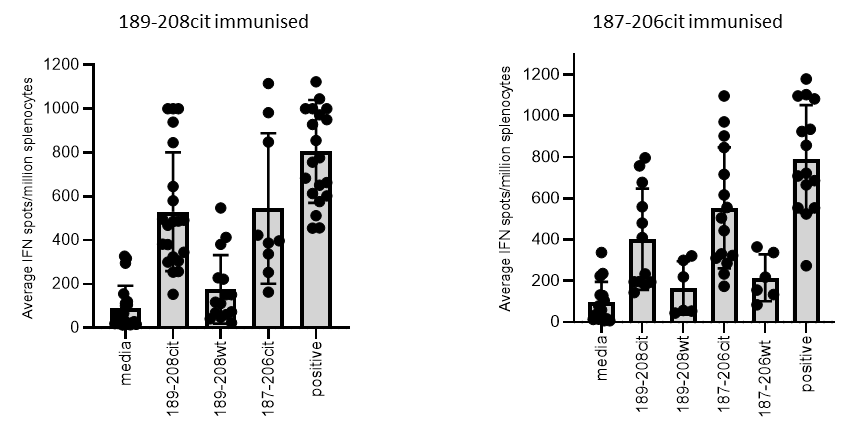


GRP78 189-208cit response cross reacts with GRP78 187-206cit sequence. HHDII/DP4 transgenic mice were immunised with GRP78 189-208cit (A) or 187-206cit (B) peptide and responses measured to both citrullinated peptides by IFNg ELISpot assay. Results show data from at least two independent experiments in which n=3.

Supplementary Figure 3.

Good ion series coverage – non-oxidized peptide – unambiguous peptide assignment


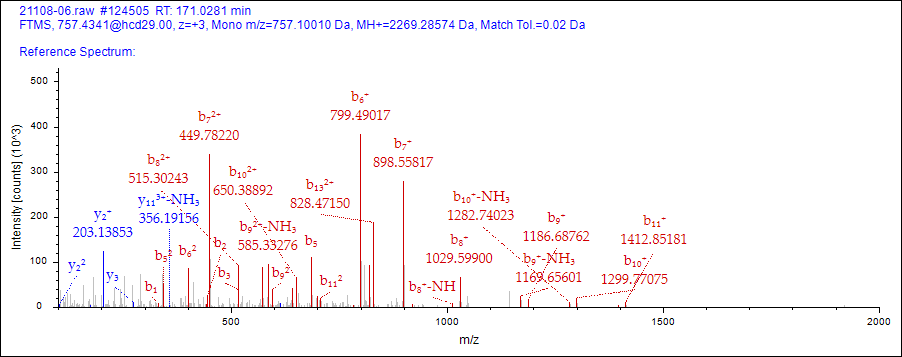


Good ion series coverage – oxidized peptide – unambiguous peptide assignment


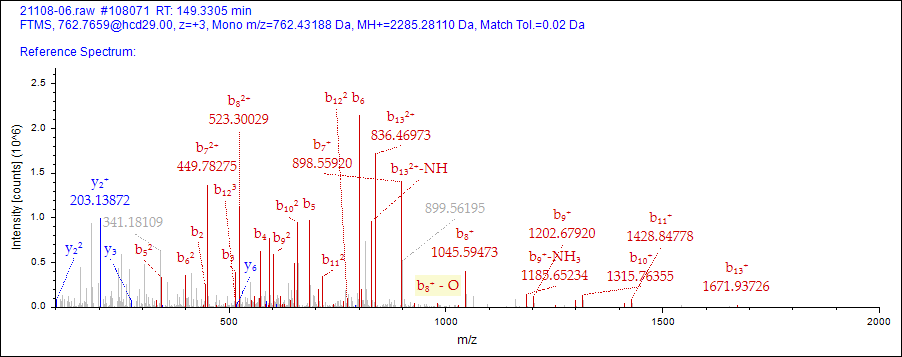


HCD fragmentation spectra from the citrullinated GRP78 189-208 peptide. The peptide was predominantly detected in the oxidized form. In both spectra (oxidized and non-oxidized forms), the b9-ion was identified, which is the site of citrullination (indicated by arrows). These are accurate mass scans, able to resolve the 1Da mass difference between the citrullinated and non-citrullinated form. In both spectra, the b-ions series is near complete ranging from the b1 to the b11 and b13-ion respectively.

Supplementary Figure 4.


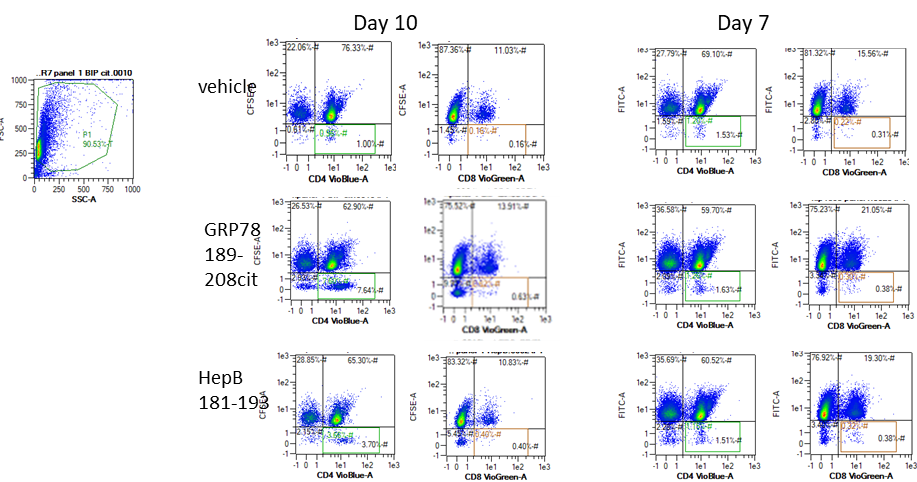


Example staining of proliferative responses at days 7 and 10 from donor BD0007 to citrullinated GRP78 189-208 peptide compared to response from a known CD4 epitope peptide from hepatitis B.

**
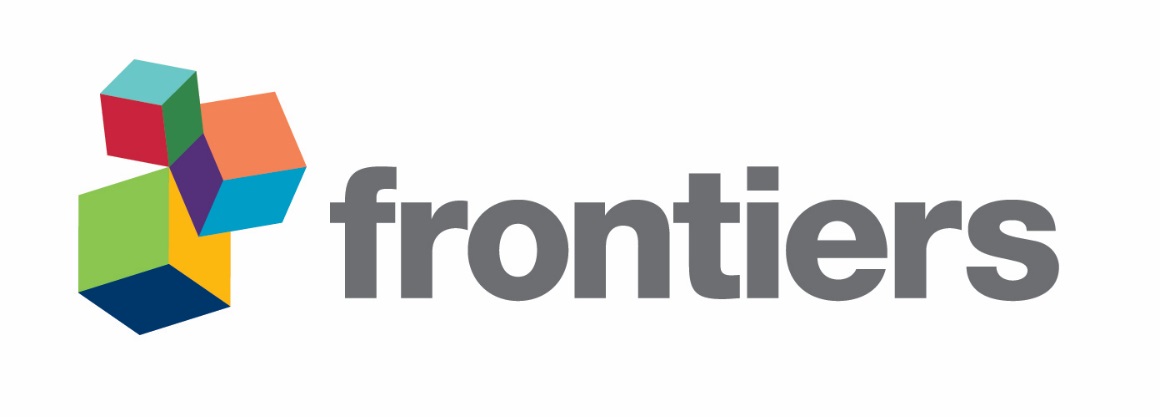
**
